# Estimation and Identification of Nonlinear Parameter of Motion Index Based on Least Squares Algorithm

**DOI:** 10.1155/2022/7383074

**Published:** 2022-05-02

**Authors:** Hong Qin

**Affiliations:** Department of Physical Education, Zhongyuan University of Technology, Zhengzhou, Henan 450007, China

## Abstract

Parameter identification is an important branch of automatic control. Due to its special function, it has been widely used in various fields, especially the modeling of complex systems or systems whose parameters are not easy to determine. With the development of control technology, the scale of the control object is getting larger and larger, which makes the calculation amount of the identification algorithm larger and larger. For the nonlinear system with complex structure, especially the nonlinear system containing the product of unknown parameters, the number of parameters of the over-parameterized identification method increases greatly, and the calculation amount of the identification algorithm also increases sharply. Therefore, a parameter estimation method with a small amount of calculation is explored. The results show that the proposed method can overcome the phenomenon of “data saturation”, thus improving the parameter identification results.

## 1. Introduction

The so-called identification is to estimate the mathematical model of the object by measuring the output response of the object under the action of human input, or the input and output data records during normal operation, and adding necessary data processing and mathematical calculations. This is because the dynamic characteristics of an object are considered to be necessarily manifested in its changing input and output data, and identification is nothing more than using mathematical methods to extract the mathematical model of the object from the data sequence. The least squares method is the most basic and most commonly used method in system parameter identification because of its simple algorithm, mature theory and strong generality [1–8].

Parameter identification is to determine a model that is closest to the external characteristics of the system from a given model class on the basis of input and output data. If researchers only pay attention to the external characteristics of the system and ignore the internal characteristics, the input and output expression can be used to describe it; if the internal characteristics of the system are also emphasized, the state space model can be used to describe it [9–15].

**Figure 1:**
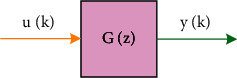
System Identification.

Generally speaking, there are two methods to establish the mathematical model of the system: excitation analysis method and system identification method. The former is to analyze and deduce the model according to the physical and chemical laws followed by the system; the latter is to obtain the model from the actual system operation and experimental data processing. As shown in Figure 1, system identification is the theory and method of calculating the mathematical model of the system from the input and output data. In addition, the system identification should also have three basic elements, namely model class, data and criteria. According to the model form, the identified system models can be divided into two categories: parametric models and non-parametric models. The so-called parametric model refers to mathematical models in the form of differential equations, difference equations, and state equations; nonparametric models refer to mathematical models with implicit parameters such as frequency response, impulse response, and transfer function [16, 17].

Parameter identification has developed rapidly as a basic subject. So far, various identification methods have been developed. Recently, many papers have studied the coupled identification methods of multivariate systems and multivariate-like systems. System identification is essentially an optimization problem. Now the common identification method is to transform the identification problem into an optimization problem by building a parameter model of the system. In the field of system identification, the theory and method of linear system identification are becoming more and more mature, such as least square identification method, hierarchical identification method, etc. However, in the actual industrial control process, there is no ideal pure linear system, it is basically a nonlinear system with complex structure and noise. Therefore, how to identify the nonlinear system and then control it more effectively has greater research value. In this paper, based on over-parameterized models and data filtering techniques, the recursive least squares identification method for outputting error-like systems of nonlinear equations is studied. The first element of identification is data, and data is also the basis of identification. The identification effect is directly affected by the data. The theory of data selection is relatively complicated. This paper does not conduct in-depth analysis and research on it. Usually, noise signals, including white noise and pink noise, are used in practice to meet the identification requirements. The identification of linear parameters uses pink noise signals [18–23].

The second element of identification is the model class, which mainly includes the least squares class, the gradient correction method, and the maximum likelihood method.

The third element of identification is the equivalence criterion, which is an index that describes the degree of conformity between the identification model and the actual system. The equivalence criterion is generally used to evaluate the model's ability to “describe” the input and output models, and since the essence of the model is its predictive ability, the equivalence criterion is also used to evaluate the model's predictive ability. There are many commonly used criteria for the equivalence criterion. In this paper, the basic cumulative error sum of squares is selected as the criterion function, and the specific form is shown in the following analysis. In identification engineering, the determination of the model is mainly based on experience to make assumptions about the characteristics of the actual object, such as whether the model of the object is linear or nonlinear, whether it is a parametric model or a non-parametric model [24–28].

Nonlinear characteristics exist widely in industrial processes, and it is difficult to obtain satisfactory results by using linearization methods to deal with nonlinear systems. Therefore, special identification methods must be studied for the special structure of nonlinear systems. For example, some literatures have proposed the least squares iteration method and gradient iteration method, recursive augmented least squares method and augmented stochastic gradient method of Hammer-stein nonlinear ARMAX system and their convergence. Auxiliary model recursive least squares method, some people have also studied and proposed the Hammerstein nonlinear system projection identification method, stochastic gradient identification method, Newton recursive identification method, Newton iteration method, etc. There are also literatures for output nonlinear moving average systems.

For the parameter identification of the motion system, the more mainstream identification methods include model adaptive identification algorithm, least square method, extended Kalman filter method, genetic algorithm, frequency sweep test and so on. Among them, the P and Q matrices of the extended Kalman filter method are difficult to determine, and are closely related to the system state; the genetic algorithm has high requirements for the initial value of the parameters to be estimated; the frequency sweep test needs to use different frequencies to excite the system, and then use the least squares method to obtain the amplitude-frequency characteristics and phase-frequency characteristics of the closed-loop system, and finally use the INVFREQS function of MTLAB to perform fitting. The accuracy is not high; in addition, the genetic algorithm and the frequency sweep test cannot achieve the online real-time estimation of parameters. Therefore, some scholars tried to use some improved methods to implement their work [29,30]. The results show that the method can accurately identify the key parameters of the system model, and the key parameter map can accurately represent the real-time dynamic characteristics of the vehicle, which lays a good foundation for the parameter estimation and stability control of vehicles. Besides, in recent years, with the continuous research on deep learning, many problems in daily life can be solved by artificial intelligence. One of the characteristics of deep learning is that programmers no longer need to continuously write programs to complete the program as in the past, but only need to build a neural network in advance, use artificial means to imitate the thinking of the human brain, and match a sufficient amount of data to simulate the training machine itself, let the machine discover and learn the data by itself.

In this paper, for the output nonlinear equation error system, the least squares identification method based on the overparameterized model is discussed. In addition, this paper also uses MATLAB software to verify the superiority of the least squares method in parameter identification, and then identifies the parameters of an unknown steering system, and obtains the equivalent moment of inertia, equivalent damping coefficient and equivalent stiffness, etc. Results show that the proposed method can overcome the phenomenon of “data saturation”, thus improving the parameter identification results.

## 2. Nonlinear Systems

The structure of nonlinear system model is various, and there is no unified mathematical expression. The most studied are nonlinear systems with simple block structure properties. The block-structured nonlinear system includes: 1) input nonlinear system (nonlinear block is located before linear block: N—L); 2) output nonlinear system (nonlinear block is located after linear block: L—N); 3) input Output nonlinear system (two nonlinear blocks sandwich a linear block: N—L—N); 4) Intermediate nonlinear system (linear dynamic subsystems at both ends sandwich a static nonlinear link: L—N—L);5) Feedback nonlinear system (static nonlinear link can be in the forward channel or in the feedback channel) and so on. Here, L stands for linear, and N stands for nonlinear. Nonlinearity in block-structured nonlinear systems usually refers to static nonlinearity.

When the input nonlinear block is a static polynomial nonlinear element and the linear block is a dynamic subsystem, such a nonlinear system is also called a Hammerstein system. When the output nonlinear block is a static polynomial nonlinear element and the linear block is a dynamic subsystem, such a nonlinear system is also called a Wiener system. The nonlinear block can be a linear combination with known basis functions and unknown parameters, or it can be hard nonlinear, such as dead-band nonlinearity, saturation nonlinearity, relay nonlinearity and so on.

The above is to define the input nonlinear system and the output nonlinear system from the composition relationship between the linear block and the nonlinear block. In fact, the input nonlinear system can also be defined from the linear and nonlinear expression relationship between the input variable and the output variable in the model. and the output nonlinear system. According to this rule, the input nonlinear system is defined as the system output y(t) is a nonlinear function of the system input u(t-i), and is a linear function of the past output y(t-i), such as the typical Hammerstein nonlinearity system. The corresponding output nonlinear system is defined as its output y(t) is a nonlinear function of the system's past output y(t-i) and a linear function of the input u(t-i), such as the nonlinear system in the literature. The input-output nonlinear system is defined as its output y(t) is not only a nonlinear function of the system input u(t-i), but also a nonlinear function of the past output y(t - i), such as the nonlinear system in the literature. The input nonlinear system includes the input nonlinear equation error system and the input nonlinear output error system. The output nonlinear system includes the output nonlinear equation error class system and the output nonlinear output error class system.

For the identification of nonlinear systems, the most common method for identifying nonlinear systems is the identification method of least squares algorithm. In the nonlinear system identification method, the input of the system is represented by a Laguerre function, the system is represented by a Hermite polynomial, and the output is represented by a Laguerre-Hermite series expansion containing a large number of unknown coefficients. This method has a strong theoretical meaning, but the amount of calculation is very large, which is not conducive to practical applications. In addition, there are many nonlinear system models involved in research, such as bilinear system model, Hammerstein model, Wiener model, nonlinear time series model and so on. For each special model, scholars all over the world have done a lot of research and proposed many identification calculations. The estimation consistency of these algorithms is also discussed. With the increasing demand of nonlinear model identification, research on nonlinear system identification problem is also more and more in-depth. Various models of nonlinear systems have been discussed by many scholars [31–35].

From the study of linear model identification to the study of nonlinear model identification, the solution method has been improved. However, because the nonlinear system itself contains a lot of uncertainty, it is difficult to deduce the identification method suitable for various nonlinear systems, so the nonlinear system identification has not yet formed a complete scientific system. In recent years, many types of identification methods have appeared, such as least squares identification method, model reference adaptation, multi-information identification method, neural network identification, iterative identification method, etc., which have opened up new ways and new ideas [36–39].

Least squares method: The advantages of this algorithm are simple and less computational, so it is well used in the identification of parameters in many control systems. The least squares method needs to linearize the model of the controlled object, so it is usually assumed that the speed change rate of the motor during the identification process is zero. Some literatures have proposed a motor parameter identification method based on the nonlinear least squares method to solve the problems of poor convergence and slow dynamic response of the parameter identification of the controlled object. This method does not require many simplified assumptions for the control algorithm. The premise is that as long as the system works under sufficient excitation conditions, the motor parameters can be updated at any time.

Model reference self-adaptation: This algorithm has been around for a short time, but the principle is simple, easy to implement and other advantages make it a wide range of applications. Its basic principle is to construct a reference model and an adjustable model. By judging whether the output errors of the two models reach the ideal value, if the conditions are met, the parameters of the adjustable model are considered to be consistent with the parameters of the reference model, and vice versa, the two parameters are not equal, then the identification process ends.

Neural network identification: This method is because the artificial neural network is similar to simulating human thinking. It abstracts and simplifies the obtained information and stores it, and then processes it to establish a relevant mathematical model, so that it has the ability to identify nonlinear systems. The advantages of this identification method are high precision and fast dynamic response. After the model is determined, the parameters of the model can be determined according to a certain identification algorithm according to the input and output data of the object.

## 3. Least Squares Parameter Estimation and Identification

The concept of parameter identification is derived from system identification, and the system is usually described by a model, and modeling is a necessary step before the identification of the system. Different types of models have their own characteristics, and the model-based identification algorithms are also very different, but they all have a basic attribute, that is, they are closely related to the input and output data of the system, and can reflect the working mechanism of the system.

The so-called model is to reduce the essential information of the system into a useful description form, which is used to represent the internal change law of the system, and is a powerful tool for analyzing the system and predicting the output. There are many different standards for classification models. According to the linearization of the model, they can be divided into linear models and nonlinear models. According to the degree of change of the models, they can be divided into dynamic models and static models.

There are three main ways to model a system, namely “white box”, “black box” and “gray box”, which are classified according to how much is known about how the system works. The “white box” modeling considers the working principle of the system in depth. The “black box” modeling treats the system to be identified as a black box, ignores the working principle of the system, adopts the method of violent solution, and uses the identification method based on the Vblterra coefficient. The model is somewhere in between. The modeling method used in this paper belongs to the “white box” modeling, and then focuses on the “white box” modeling. “white-box” modeling, also known as mechanistic modeling, is a theory-based approach to modeling.

The working principle of the system is used to establish a model of the system, which can generally only be used for systems with a clear working mechanism. In addition, “white-box” modeling often requires a reasonable simplification of the system under study, such as ignoring noise, analyzing variables as constants, etc. Otherwise, the problem is too complicated, which will greatly increase the difficulty of identification, and the mechanism of the system must be deterministic and derivable.

For the parameter model identification structure, the task of system identification is parameter estimation, that is, using input and output data to estimate these parameters and establish a mathematical model of the system. The most commonly used methods in parameter estimation are the least squares method, the error prediction estimation method, the auxiliary variable method, and the neural network method.

**Figure 2:**
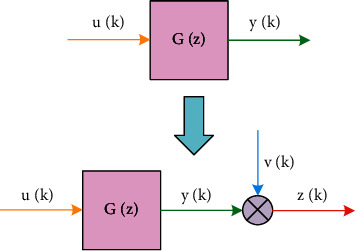
Single input/single output.

Because the least squares method is easy to understand and master, the identification algorithm based on the principle of the least squares method is relatively simple in implementation, and does not require knowledge of mathematical statistics, making the least squares method widely used in the field of system identification, but it also has certain When the system noise is colored noise, the least squares method cannot give unbiased consistent estimates. In this paper, the motion control system model is used for parameter identification. Set up a “black box” structure of a SISO (single input/single output) process, as shown in Figure 2:

The so-called least-squares recursive algorithm is that when identifying the system parameters, every time a new observation data is obtained, on the basis of the previous estimation, the newly introduced observation data is used to continuously revise the results of the previous estimation. Thus, new parameter estimates are obtained recursively.

Compared with the least squares one-time completion algorithm, it can not only reduce the amount of calculation and storage, but also realize online real-time identification. However, it has three defects: (1) When the model noise is colored noise, the least squares parameter estimation is not unbiased and consistent; The estimated value and accuracy of the unknown parameters cannot reach the expected results, and even make the identification results worse; 3. The least squares parameter estimation cannot track the changes of time-varying parameters, that is, it is not suitable for the parameter identification of time-varying processes. In order to overcome the phenomenon of data saturation and weaken the influence of old observation data on the estimation of unknown parameters, two improved algorithms of least squares are used, namely fading memory and limited memory least squares recursive algorithm.

The basic idea of the fading memory recursive least squares method is to give different weights to the sampled data, that is, to add a forgetting factor to the old data to reduce the amount of information provided by the old data and increase the amount of information of the new data. This not only considers the role of historical data, but also focuses on new information for parameter estimation.

For the conventional least squares method, as the gain matrix K(k) is close to zero, the parameter estimates change very little, indicating that the algorithm basically loses the correction ability, and the recursive calculation will not further improve the identification accuracy. However, for the fading memory least squares method, the parameter estimates are always fluctuating, indicating that the information provided by the new data is still working.

The transfer function of the system is:

(1) $$\begin{array}{c} G\left(z\right)=\frac{b_{1}z^{-1}+b_{2}z^{-2}+...+b_{n}z^{-n}}{1+a_{1}z^{-1}+a_{2}z^{-2}+...+a_{n}z^{-n}} \end{array}$$

Then

(2) $$\begin{array}{c} y\left(k\right)=-\sum_{i=1}^{n}a_{i}y\left(k-i\right)+\sum_{i=1}^{n}b_{i}u\left(k-i\right) \end{array}$$

**Figure 3:**
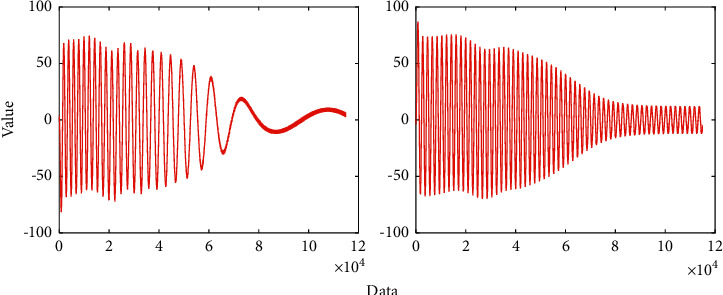
Predicted value.

Among them, *y* is the kth true value of the system output, and *u* is the *k*th input value of the system. The predicted value is shown in figure 3. The proposed method exhibits a better performance. As can be seen, the flotation is lower while it still keeps the value as the original one.

If considering that the identified system or observation information contains noise, the final output is:

(3) $$\begin{array}{c} z\left(k\right)=-\sum_{i=1}^{n}a_{i}y\left(k-i\right)+\sum_{i=1}^{n}b_{i}u\left(k-i\right)+v\left(k\right) \end{array}$$

Where, *z* is the kth observation of the system output, *v* is the random noise with mean 0. If defined:

(4) $$\begin{array}{c} h\left(k\right)=\left[-y\left(k-1\right),-y\left(k-2\right),...,-y\left(k-n\right),u\left(k-1\right),u\left(k-2\right),...,u\left(k-n\right)\right] \end{array}$$

(5) $$\begin{array}{c} \theta ={\left[a_{1},a_{2},...,a_{n},b_{1},b_{2},...,b_{n}\right]}^{T} \end{array}$$

where, *θ* is the parameter to be estimated.

So, *z* can be expressed as:

(6) $$\begin{array}{c} z\left(k\right)=h\left(k\right)\theta +v\left(k\right) \end{array}$$

Let

(7) $$\begin{array}{c} k=1,2,...m \end{array}$$

Then

(8) $$\begin{array}{c} z_{m}=\left[\begin{array}{c} z\left(1\right)\\ z\left(2\right)\\ .\\ .\\ .\\ z\left(m\right) \end{array}\right] \end{array}$$

(9) $$\begin{array}{c} H_{m}=\left[\begin{array}{c} h\left(1\right)\\ h\left(2\right)\\ .\\ .\\ .\\ h\left(m\right) \end{array}\right] \end{array}$$

The idea of least squares is to find an estimate of *θ* such that the sum of squares of the difference between the z of each measurement and the estimate of the measurement determined by the estimate is the smallest, that is:

(10) $$\begin{array}{c} J\left(\hat{\theta }\right)={\left(Z_{m}-H_{m}\hat{\theta }\right)}^{T}\left(Z_{m}-H_{m}\hat{\theta }\right)=\min \end{array}$$

(11) $$\begin{array}{c} \frac{\partial J}{\partial \theta }=-2H_{m}^{T}\left(Z_{m}-H_{m}\hat{\theta }\right)=0 \end{array}$$

Then,

(12) $$\begin{array}{c} H_{m}^{T}H_{m}\hat{\theta }=H_{m}^{T}Z_{m} \end{array}$$

**Figure 4:**
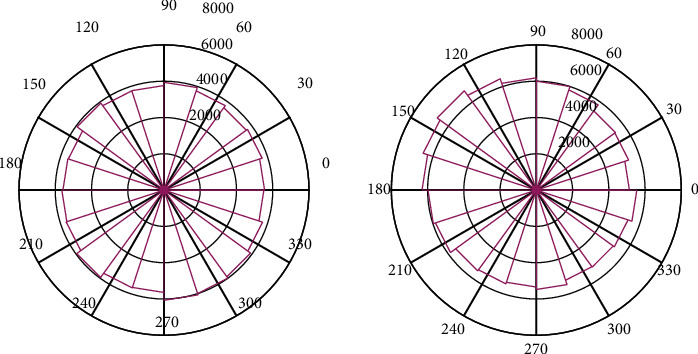
Radar value.

Although the least squares estimation cannot satisfy every equation in the measurement equation and make each equation have deviations, it minimizes the squared sum of the deviations of all equations, takes into account the approximation of all equations, and minimizes the overall error. This is beneficial to suppress measurement errors.  Figure 4 shows the radar value. Mathematical models are the basis for studying the laws of motion of all things, and parameter identification is the theory and method for studying the establishment of mathematical models of systems.

## 4. Simulation Analysis

**Figure 5:**
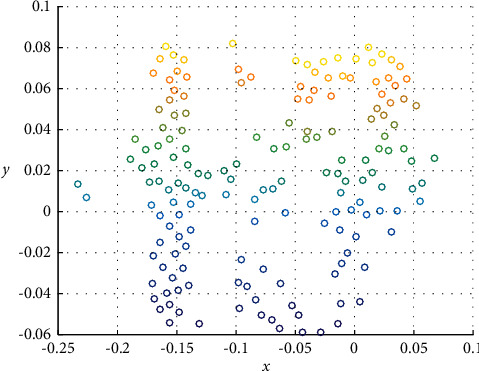
x, y variation.

If the structure of the established system mathematical model is selected correctly, the accuracy of the model parameter identification will directly depend on the system input signal. Therefore, selecting a reasonable input signal is one of the key factors to ensure that the ideal identification result can be obtained. Theoretical analysis shows that selecting white noise as the input signal for identification can obtain better identification results, but this is almost difficult to achieve in engineering, because actual industrial equipment cannot act according to the changing law of white noise. Replace the white noise signal with an inverse M-sequence that approximates white noise. The x, y variation is shown in figure 5.

**Figure 6:**
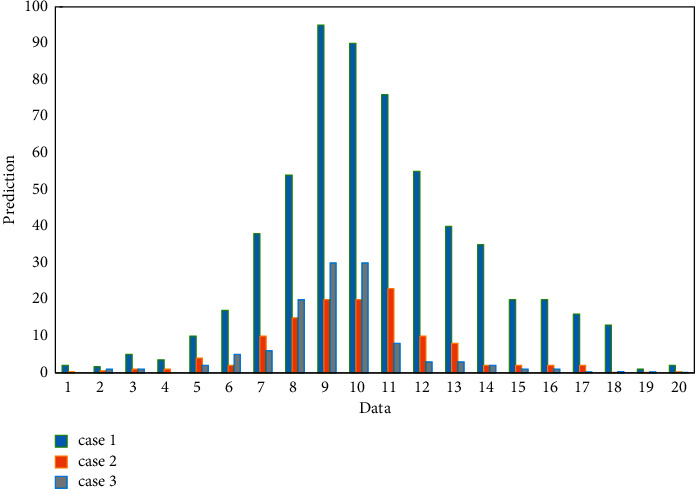
Predicted data.

Spectral analysis shows that the M-sequence usually contains a DC component, which may cause a “net perturbation” in the identification system, which is usually undesirable. The inverse M-sequence can overcome this shortcoming and is a more ideal pseudo-random code sequence than the M-sequence. Let M(k) be an M sequence with a period of N p bits and an element value of 0 or 1, and S(k) a square wave sequence with a period of 2 bits and an element value of 0 or 1 in turn. Perform bitwise XOR operation, and the obtained composite sequence is an inverse M sequence with a period of bit and an element value of 0 or 1. The predicted data is shown in Figure 6.

**Figure 7:**
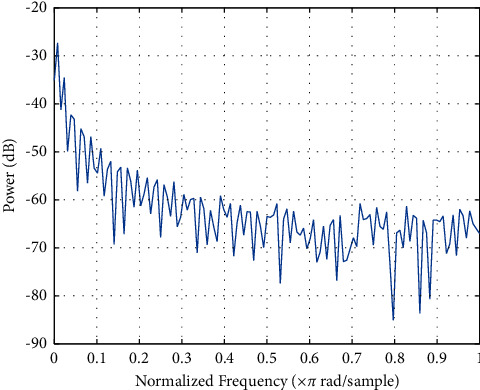
Normalized frequency.

Replace the logical value “0” or “1” of the above inverse M-sequence with -1 or 1 respectively, and the mean value of the inverse M-sequence is 0 at this time. Although the inverse M-sequence is the result of a simple combination of the M-sequence and the square wave sequence, its properties are superior to the M-sequence, making it more widely used in the field of identification. The normalized frequency is shown in figure 7.

It can be seen from Table 1 that the estimated relative errors of the first three parameters are all within 0.2%, while the last parameter can be accurately estimated to the thousandths by the recursive least squares method due to its very small value. Also controlled within 4%. And with the continuous increase of the data taken, the results of the system identification will also improve. This is an advantage of the recursive least squares method, which can improve the previously identified results according to the continuously updated data, so that the identified results are closer to the true value.

**Figure 8:**
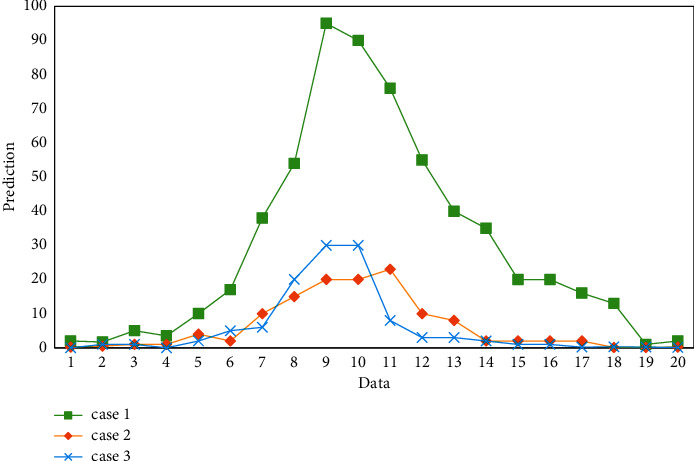
The prediction.

In this simulation, 10 000 sets of data are collected, as shown in Table 1, and the change curve of each parameter identification can be observed, where a 1 is the estimated curve of -39/18, a 2 is the estimated curve of 16/19, and a 3 is -5/ 9 is the estimated curve, b 0 is the estimated curve of 1/180, and k is the simulation step size. The prediction is shown in figure 8.

**Figure 9:**
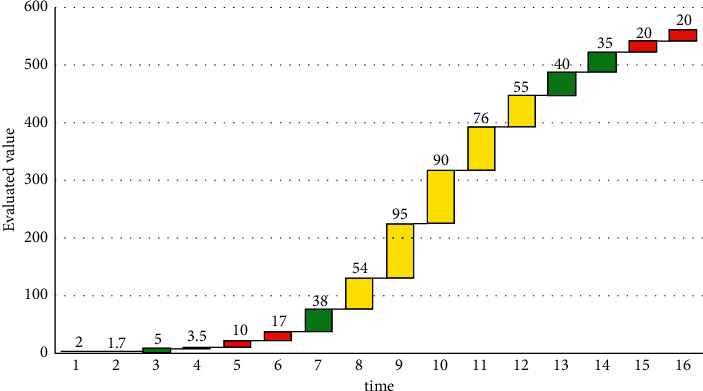
Evaluated value.

To sum up, it can be concluded that the recursive least squares method can accurately identify the parameters, and the identification results are ideal. The evaluated value is shown in figure 9.

## 5. Conclusion

Parameter identification is an important branch of automatic control. Due to its special function, it has been widely used in various fields, especially the modeling of complex systems or systems whose parameters are not easy to determine. With the development of control technology, the scale of the control object is getting larger and larger, which makes the calculation amount of the identification algorithm larger and larger. For the nonlinear system with complex structure, especially the nonlinear system containing the product of unknown parameters, the number of parameters of the over-parameterized identification method increases greatly, and the calculation amount of the identification algorithm also increases sharply. Therefore, a parameter estimation method with a small amount of calculation is explored. For the output nonlinear equation error system, the least squares identification method based on the overparameterized model is discussed. And proposed a least squares identification method based on model decomposition and a least squares identification method based on data filtering. In addition, this paper also uses MATLAB software to verify the superiority of the least squares method in parameter identification, and then identifies the parameters of an unknown steering system's kinematic index, and obtains the equivalent moment of inertia, equivalent damping coefficient and equivalent stiffness, etc. motion parameters, and verify the identified parameters. Simulation results show that the proposed method can overcome the phenomenon of “data saturation”, thereby improving the parameter identification results.

## Figures and Tables

**Table 1:** Estimated relative errors

Item	a1	a2	a3	b0
real	-2.1667	1.7778	0.5556	0.0055
prediction	-2.1681	1.7799	0.5553	0.0057
error	0.065	0.12	0.054	3.64

## Data Availability

The data used to support the findings of this study are available from the corresponding author upon request.
